# Endoscopic shelf acetabuloplasty can improve clinical outcomes and achieve return to sports-related activity in active patients with hip dysplasia

**DOI:** 10.1007/s00167-017-4787-0

**Published:** 2017-11-28

**Authors:** Soshi Uchida, Akihisa Hatakeyama, Shiho Kanezaki, Hajime Utsunomiya, Hitoshi Suzuki, Toshiharu Mori, Angela Chang, Dean K. Matsuda, Akinori Sakai

**Affiliations:** 1 Department of Orthopaedic Surgery, Faculty of Medicine, Wakamatsu Hospital of the University of Occupational and Environmental Health, 1-17-1, Hamamachi, Wakamatsu, Kitakyushu, Fukuoka 808-0024 Japan; 2 Department of Orthopaedic Surgery, Faculty of Medicine, University of Occupational and Environmental Health Japan, Kitakyushu, Japan; 3 DISC Sports and Spine, Marina del Rey, CA USA; 4 Steadman Philippon Research Institute, Vail, USA

**Keywords:** Hip arthroscopy, Developmental dysplasia of the hip, Acetabular labral tear, Shelf acetabuloplasty, Return to sport

## Abstract

**Purpose:**

To investigate clinical outcomes and return to sports-related activity following endoscopic shelf acetabuloplasty combined with labral repair in the treatment of the active patients with developmental dysplasia of the hip (DDH).

**Methods:**

Between 2011 and 2013, 32 patients (36 hips; 11 males and 21 females; 11 right 17 left 4 bilateral; median age 28.5, range 12–51 years), who underwent endoscopic shelf acetabuloplasty combined with labral repair and met the inclusion criteria were enrolled in this study. There was a minimum follow-up of 2 years (average 32.3 ± 3 months, range 24–48 months). Patient-reported outcome (PRO) scores including the modified Harris Hip Score (MHHS) and Non-Arthritis Hip Score (NAHS) were obtained preoperatively and at final follow-up for the assessment of surgical outcomes.

**Results:**

The mean MHHS significantly improved from 68.4 ± 14.3 (range 23.1–95.7) preoperatively to 94.5 ± 8.5 (range 66–100) at final follow-up (*p* = 0.001). Similarly, the NAHS also significantly improved from 51.3 ± 11.9 (range 23–76) preoperatively to 73.0 ± 7.4 (range 44–80) at final follow-up (*p* = 0.001). The mean LCE angle significantly increased postoperatively but partially decreased at final follow-up (mean preoperative versus postoperative versus final follow-up: 16.0 range 5–24, versus 40.1 range 27–58, versus 30.1 range 20–41. *p* = 0.001, respectively). There were 3 patients who returned to a higher activity level, 20 patients who returned to the same activity level, and 6 patients who returned to a lower activity level. The mean period from surgery to return to play was 9.0 ± 3.5 months (range 5–18).

**Conclusion:**

Endoscopic shelf acetabuloplasty provides promising clinical outcomes and return to sports-related activity for active patients with DDH.

**Level of evidence:**

Level IV.

## Introduction

Developmental dysplasia of the hip (DDH) is a rare cause of hip pain in the US but is one of the most common sources of hip pain in young active patients in the Asian population. Active patients especially athletes with DDH typically experience frequent groin and lateral hip pain associated with intra-articular pathology including acetabular labral tear and cartilage damage [25]. There is disparate evidence for and against hip arthroscopy in the setting of DDH. Emerging evidence suggest that Cam deformities often co-exist with DDH [30]. While the mildest forms of DDH (borderline) may respond favorably to isolated hip arthroscopy with femoroplasty and labral and capsular repairs, mild to moderate DDH may have less successful outcomes in the short-term [7, 8, 15, 19] Previous studies established hip arthroscopy as a beneficial technique for treating mild DDH [3, 17, 33]; however, the recent literature reports a high reoperation rate and lateral migration with conversion to total hip arthroplasty [13, 24]. One study advise against performing hip arthroscopy for DDH when a broken Shenton line, a femoral neck shaft angle > 140°, and lateral center edge (LCE) angle < 19° are observed, or when severe cartilage damage is present at the time of surgery [30].

Rotational acetabular osteotomy (RAO) and periacetabular osteotomy (PAO) are beneficial procedures for treating patients with DDH, especially moderate and severe forms [9, 20]. However, high-demand athletes with DDH are not good candidates for these conventional approaches including PAO or RAO because of prolonged postoperative rehabilitation and unestablished ability to return to sport [5, 6]. Uchida et al. described new shelf acetabuloplasty endoscopic technique with arthroscopic chondrolabral and capsular reparative surgery to better access and address osseous anterolateral coverage [31]. This minimally invasive procedure is associated with less postoperative morbidity and has a greater potential to enable athletes to return to high functioning activities. It was hypothesized that symptomatic active patients with hip dysplasia can benefit from endoscopic shelf acetabuloplasty. The purpose of this study was to investigate the clinical outcomes and return to sports activity after endoscopic shelf acetabuloplasty combined with Cam osteoplasty with labral and capsular repairs.

If this surgery provides clinical benefit to athletic patients that might otherwise require open RAO/PAO, it could establish endoscopic shelf acetabuloplasty as a viable less invasive surgery extending to the active patient population.

## Materials and methods

**Fig. 1:**
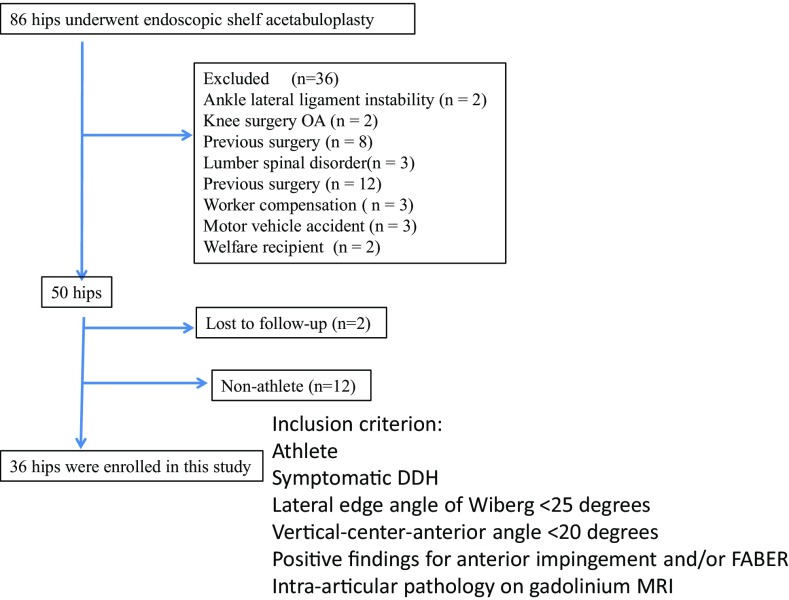
Flowchart showing the recruitment of patients with developmental dysplasia of the hip undergoing endoscopic shelf acetabuloplasty in this study

**Fig. 2:**
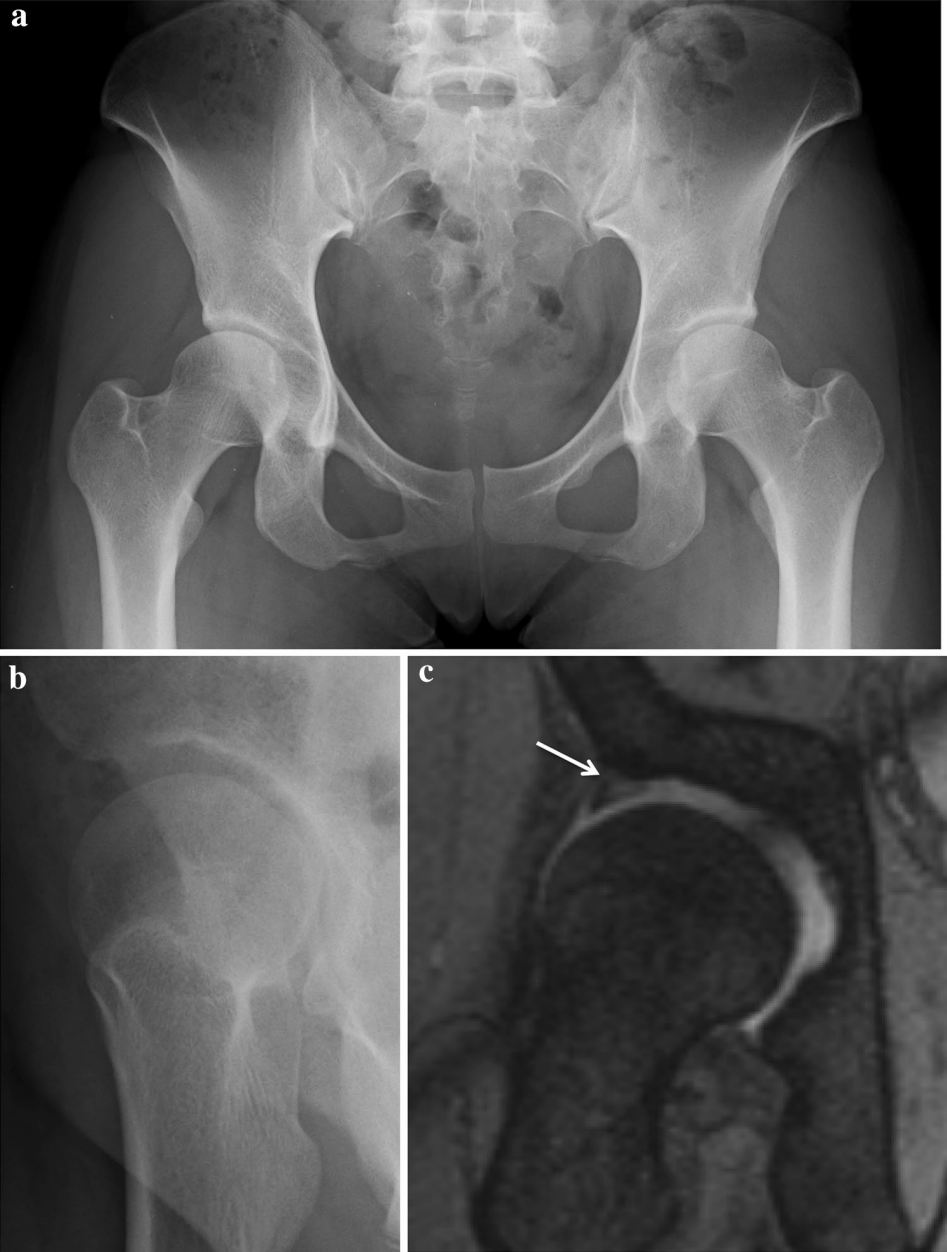
A 17-year-old speed skater presented to us with a 6 month history of right hip pain. **a** Diagnostic preoperative pelvic AP radiograph shows DDH. The center edge angle is 16° and the sharp angle is 49°. **b** Preoperative false profile view also shows anterior shallowness of acetabulum. The vertical–center–anterior (VCA) angle is 18°. **c** T2-weighted magnetic resonance imaging showing an area of high at acetabular labrum, suggestive of a possible acetabular labral tear

The medical records of 80 patients (86 hips) who underwent endoscopic shelf acetabuloplasty by a single surgeon (S.U) between 2011 and 2013 were retrospectively reviewed. Endoscopic shelf acetabuloplasty were indicated for symptomatic DDH (Fig. 1), defined as recalcitrant pain in hip and/or groin unresponsive to conservative treatment for a minimum of 3 months (e.g., NSAIDs, modification of painful sports and/or hip flexion-related activities, physical therapy), a lateral center edge (LCE) angle of Wiberg of less than 25° [32] on pelvic anterior–posterior view (AP) and/or vertical–center–anterior (VCA) angle of less than 20° on false profile view [16] (Fig. 2a, b), positive provocative maneuvers (anterior impingement and/or FABER tests), and intra-articular pathology including acetabular labral tears as detected by a gadolinium-enhanced magnetic resonance (MR) arthrogram (Fig. 2c). Contraindications for hip arthroscopy in DDH included osteoarthritis (Tönnis grade II and III), Legg Calve Perthes disease, and lateral migration of the femoral head. A total of 36 hips were excluded; 13 of these hips belonged to non-athletes. Patients with either ankle lateral ligament instability (*n* = 2), knee osteoarthritis (Kellgren and Lawrence classification osteoarthritis grade 3 and 4) (*n* = 2), history of a previous hip surgery (*n* = 8), lumbar spine disorder (*n* = 3), worker compensation (*n* = 3), history of motor vehicle accident (*n* = 3) or Welfare recipients (*n* = 2) were excluded. 12 non-athlete hips were excluded. Two patients (two hips) were lost to follow-up.

Thirty-two patients (36 hips, 11 males and 21 females), who participated in competitive sports, met our inclusion criteria and were enrolled in this study (Fig. 1). Patients were defined as active patients based on the University of California-Los Angeles Activity Scale (UCLA-AS) score of $\ge$7. We also defined active patients as those who play at least one organized sport in formal competition. There were three in baseball, three in dance, two in badminton, two in ballet, two in track and field, two in climbing, two in Judo, two in rhythmic gymnastics, two in basketball, two in running and one each in table tennis, swimming, karate, cycling, rugby, kyudo, volleyball and baton twirling. All our patients had adequate self-reported participation in sports that met both criteria at the time of injury.

Surgical outcomes were obtained and assessed for 32 patients (36 hips, 11 males and 21 females 15 right hips and 13 left 4 bilateral hips). The mean BMI at the time of surgery was 21.8 (16.9–26.4).

The median age at the time of surgery was 28.5 years old (range 12–51 years). There was a minimum follow-up of 2 years (average 32.3 ± 3 months, range from 24 to 48) (Tables 1, 2).

**Table 1:**
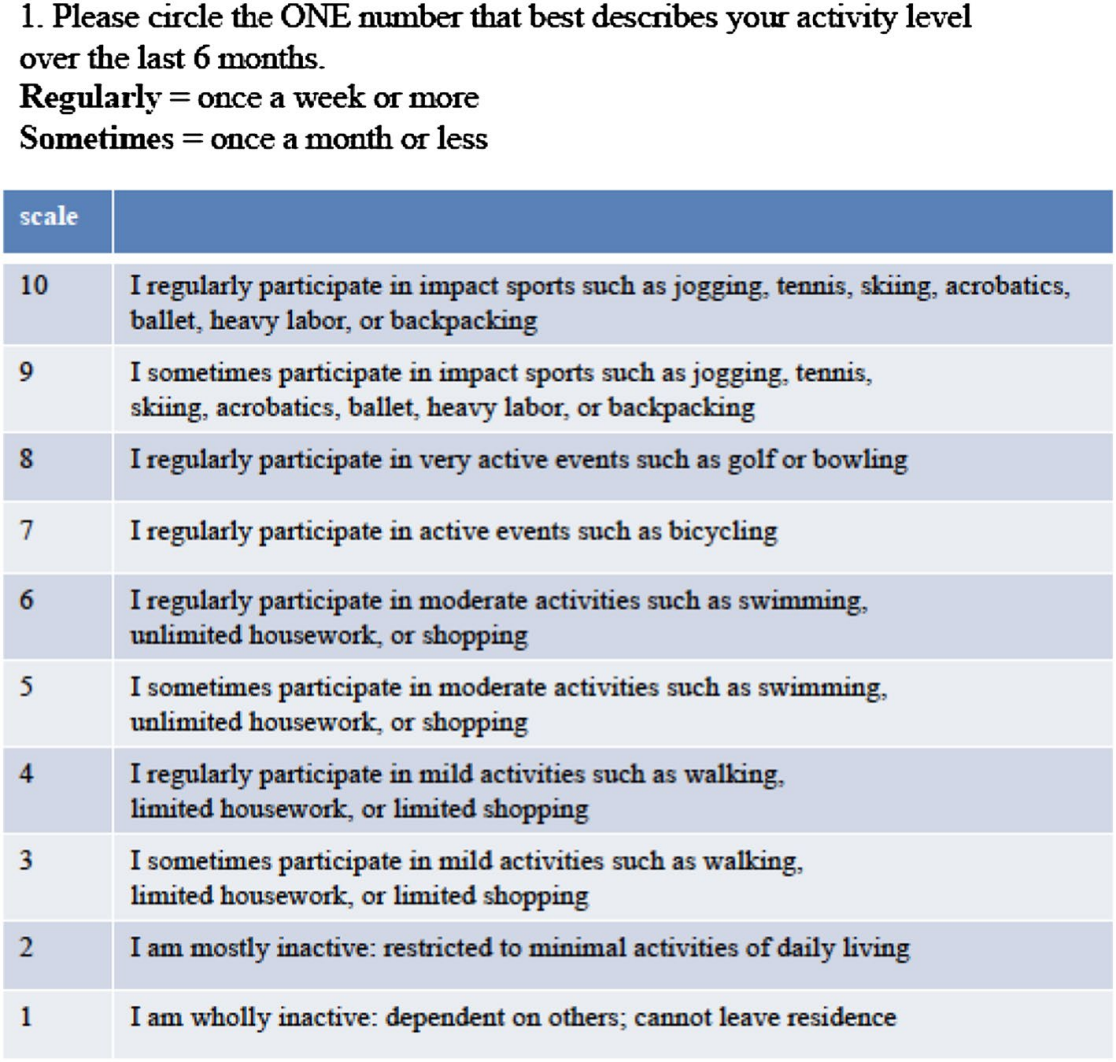
UCLA activity scale

**Table 2:** Patient demographic characteristic of study cohort

	Data
Patients eligible for current study (*n*)	32
Number of hips	36
Age mean (range) (years)	28.5 (12–51)
Gender	
Male	11 (34%)
Female	21 (66%)
Side	
Right	15 (46.8%)
Left	13 (40.6%)
Bilateral	4 (12.5%)
BMI mean (range) (kg/m^2^)	21.8 (16.9–26.4)

*BMI* body mass index

### Radiographic evaluation

The preoperative radiographs of the 32 patients (36 hips) were assessed. All radiographic measurements were manually assessed by two authors using picture archiving and communication system (PACS) A.K and S.K. We determined the LCE angle, the Tönnis angle, the femoral neck shaft (FNS) angle, the presence or absence of a broken Shenton line on pelvic AP view, the VCA angle on false profile view, and alpha angle on cross-table lateral view or modified Dunn view (Fig. 2a–c) [16, 22, 32].

The alpha angle reflects the amount of Cam deformity and is measured by drawing a line parallel to the femoral neck to the center of the femoral head and a concentric circle is then drawn around the femoral head. A second line is drawn from the center of the concentric circle around the femoral head to the point at which the femoral head–neck junction falls outside of the concentric circle. The angle subtended between these lines is the alpha angle. A Cam deformity is defined by an alpha angle greater than 55° on plain radiograph (Fig. 2c) [22]. All preoperative and yearly postoperative radiographs for osteoarthritic changes were evaluated using the Tönnis osteoarthritis grade classification system [29].

The inter-observer and intra-observer reproducibility of these radiographic parameters were investigated. For inter-observer reliability, two hip surgeons (A.K and S.K) blinded to the clinical data and details of radiology reports, independently measured each radiograph. For intra-observer reliability, one hip surgeon (A.K) individually measured radiographs at three separate times, with at least 1 week between measurements. Inter-class correlation coefficients (ICCs) and corresponding 95% confidence intervals (CIs) were calculated to quantify inter-observer and intra-observer reliability for continuous variables. The weighted kappa value was used to determine agreement between two surgeon observation for a broken Shenton line and Tönnis osteoarthritis classification for hip dysplasia. Kappa values and ICCs of 1.0 are indicative of perfect agreement. The strength of agreement was interpreted as follows: ICC greater than 0.8 indicated almost perfect agreement; ICCs of 0.61–0.80, substantial agreement; ICCs of 0.41–0.60, moderate agreement; and ICCs of 0.21–0.40, fair agreement. Based on the standards for the kappa statistic proposed by Landis and Koch, our measurements were in substantial agreement to the Tönnis osteoarthritis classification system [14]. At the preoperative examination, the mean LCE angle was 16° ± 4.8° (range 5–24), the mean Sharp angle was 47.9° ± 3.7° (range 43–58), the mean Tönnis angle was 15.3° ± 6.3° (range 1.6–34.0), the mean alpha angle was 65° ± 16.8° (range 47–98), and mean VCA angle was 18° ± 6.8° (range − 3 to 24).

### Surgical technique

**Fig. 3:**
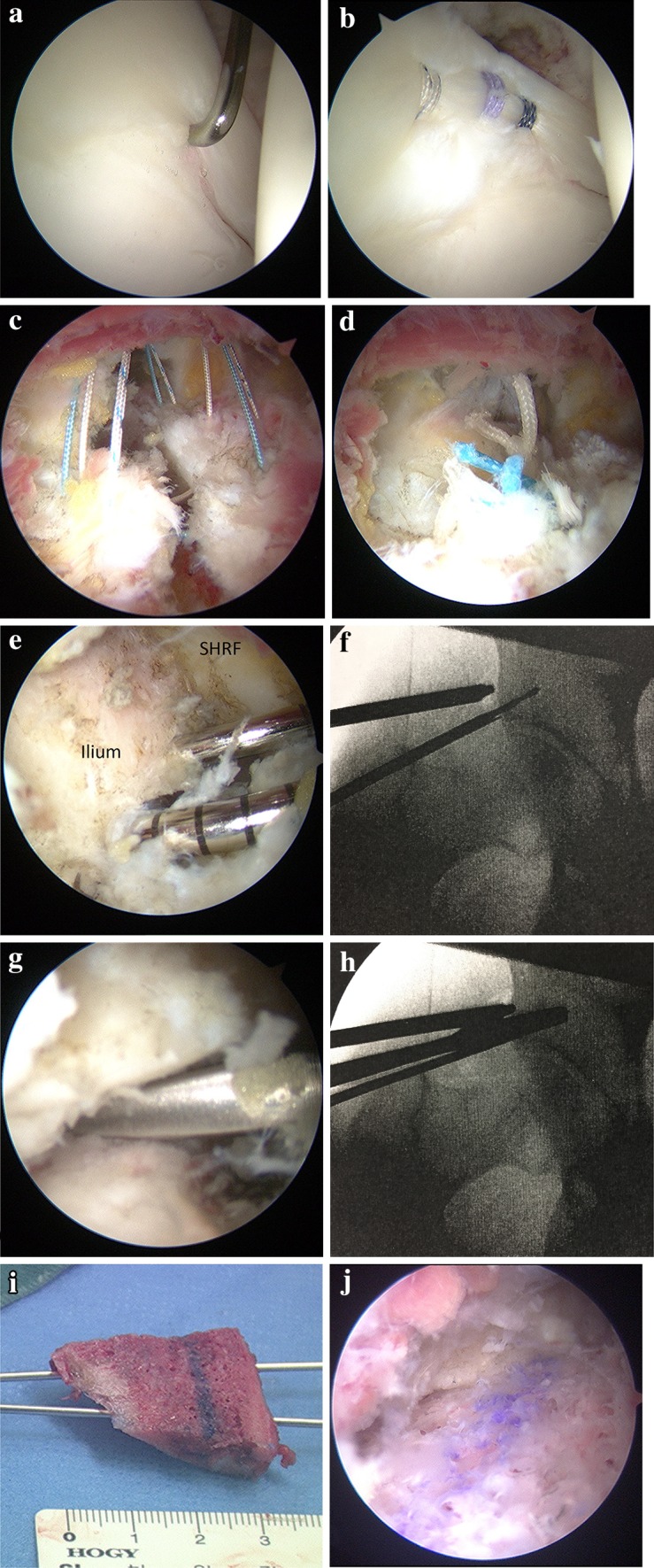
Endoscopic shelf acetabuloplasty. **a** Supine arthroscopic view from the anterolateral portal (ALP) showing an anterior superior labral tear. **b** Midsubstance labral repair is visualized from the ALP. **c, d** Arthroscopic view from the ALP showing capsular closure using strong suture passed via the mid-anterior portal (MAP) and the proximal mid-anterior portal (PMAP). **e, f** Two 2.4-mm guide wires were introduced through the MAP under fluoroscopy. **g, h** Osteotome was utilized to make the shelf slot along with 2.4 mm guide wires. **i** Free bone graft harvested from ipsilateral iliac crest, with 2 parallel 1.5-mm Kirschner wires. **j** The free bone autograft was inserted into the slot through the guidewires with press-fit fixation

Supine hip arthroscopy was performed on a traction table under general anesthesia. Anterolateral, mid-anterior and proximal mid-anterior portals (ALP, MAP and PMAP) were created. Inter-portal capsulotomy was performed. Intra-articular pathologies including acetabular chondrolabral damage and femoral head chondral damage were assessed and documented (Fig. 3a). Microfracture chondroplasty was performed if ICRS grade III or IV chondral defects were present.

Next, unstable labral tears were addressed with midsubstance repair following conservative rim trimming using a motorized burr to create a bleeding bone surface. Midsubstance labral repair was performed using bioabsorbable suture anchors (OsteoRaptor, Smith & Nephew, Andover, MA or Gryphon BR, Depuy Mitek Sports, Raynam MA) with knots tied on the capsular side of the labrum [27] (Fig. 3b). Arthroscopic dynamic examination was performed to assess for Cam impingement. When necessary, Cam osteochondroplasty using a motorized round burr was performed. Following Cam impingement evaluation and repair, capsular closure was performed with the hip at 40° of flexion via the MAP and PMAP. Typically three to five stitches with extra-capsular knots were used for capsular plication (Fig. 2c, d) [31].

Endoscopic shelf acetabuloplasty was then performed as described previously [31]. A 30° arthroscope was positioned into the extra-capsular space under the guidance with fluoroscopic imaging. After identifying the straight head and reflected head of the rectus femoris and debriding the latter with a shaver and radiofrequency ablator, two parallel 2.4-mm guide-wires were introduced using the drill guide through the MAP, along the anterior acetabular rim adjacent to the capsule (Fig. 3e, f). The slot was enlarged with the use of 10-mm osteotome to measure approximately 5–6 mm in height, 25 mm in width and at least 20 mm in depth. The optimum width and depth were confirmed using a custom-made dilator (Fig. 3g, h). Autologous tri-cortical bone graft (tri-cortical) harvested from the ipsilateral iliac crest (Fig. 3i). Two 1.5 mm Kirshner wires were introduced in 1.8 mm-diameter drill holes, helping to control the graft position during endoscopic insertion into the aforementioned anterolateral periacetabular slot (Fig. 3i). Finally, the free bone graft was secured into the appropriate position, with cortical surface facing the femoral head in intimate contact with the intervening capsule, using a press-fit technique with a cannulated bone tamp (Smith & Nephew, Japan) (Fig. 3j). Corticocancellous bone chips were inserted above the new shelf under endoscopic guidance.

### Postoperative management and rehabilitation protocol

Patients were instructed to remain non-weight bearing for the first 3 weeks. During this time, the patients were provided a brace for 3 weeks to limit hip ROM. Passive ROM exercises were initiated during the first week by physical therapists. Circumduction exercises were performed for the first 2 weeks to avoid adhesive capsulitis. Patients were allowed to participate in normal activities of daily living over a 2-month postoperative period. Patients resumed to physical activity once maximized ROM and stable gait were achieved over a 3-month postoperative period.

Intra-articular pathologies, including labral tearing, ligamentum teres and chondral damage were also evaluated according to the Multicenter Arthroscopy of the Hip Outcomes Research Network (MAHORN) classification of acetabular rim lesions, as well as cartilage damage at femoral head according to the international cartilage research society (ICRS) classification [26].

Patients completed detailed patient-reported outcome (PRO) scores including the modified Harris Hip Score (MHHS; of a possible 100 points) [2], the Non-Arthritis Hip Score (NAHS; of a possible 100 points) [4] and International Hip Outcome tool (iHot-12; of a possible 100) [11].

Patient’s athletic activity was also quantified according to the UCLA-AS guidelines (Table 1) [34].

The institutional review board (IRB) approved the study and all study subjects were provided informed consent (University of Occupational and Environmental Health, Approve number: H28-095).

### Statistical analysis

Statistical analyses about clinical outcomes and radiographic parameters of all 32 patients were performed using the SPSS (version 13, SPSS Inc., Chicago, IL, USA) software package. Power analysis was conducted using G*Power (ver. 3.1, Universität Düsseldorf, Düsseldorf, Germany). An *α* error was set 0.05, and a 1 − *β* error was set 0.80. Wilcoxon signed-rank test was used to compare paired nonparametric data and the Mann–Whitney *U* test was used to analyze nonparametric paired data. A *p* value of 0.05 or less was considered statistically significant.

Power analysis was performed for the initial six patients comparing preoperative to postoperative NHS. Effect size and sample size were calculated as 2.19 and 5 [actual power (1 − *β*): 0.93], respectively. Post hoc power analysis showed the actual power of this study was 1.0.

## Results

### Arthroscopic findings and operative procedures performed

Arthroscopic findings including labral tear, ligamentum teres injury and cartilage damage at the time of surgery are detailed in Table 3. The arthroscopic procedures performed in this study cohort are shown in Table 4. With the respect to labral management, 97% (35 hips) of procedures underwent a labral repair and 3% (1 hip) underwent labral reconstruction using iliotibial band autograft. All patients underwent capsular plication. 78% (28 hips) required cam osteochondroplasty. 3% (1 hip) of procedures required microfracture. 3% (1 hip) removal of loose bodies.

**Table 3:** Arthroscopic findings of this study cohort

	Data
Labral tear	
Complete	4 (11%)
Frayed	2 (6%)
Partial	29 ( 80.5%)
Combined	1 (3%)
Ligamentum teres	
Intact	26 (72%)
Synovitis	7 (20%)
Partial	3 (8%)
Complete	0 (0%)
Cartilage damage of femoral head (ICRS)	
Zero	30 (83%)
I	2 (6%)
II	3 (8%)
III	1 (3%)
IV	0 (0%)
Cartilage damage of acetabular rim (MAHORN)	
I	4 (11%)
II	18 (50%)
III	9 (25%)
IV	4 (11%)
V	1 (3%)

*LCE* center edge, *VCA* vertical–center–anterior, *LCE* lateral center edge

**Table 4:** Arthroscopic procedures of study cohort

	Data
Labral management	
Labral repair	35 (97%)
Labral selective debridement	0 (0%)
Labral reconstruction	1 (3%)
Total	36 (100%)
Capsular management	
Capsular plication	36 (100%)
Left in situ	0 (0%)
Total	36 (100%)
Ligamentum teres debridement	1 (3%)
Cam osteochondroplasty	28 (78%)
Trochanteric bursectomy	0 (0%)
Loose body removal	1 (3%)
Microfracture	1 (3%)

### Radiographic changes

**Fig. 4:**
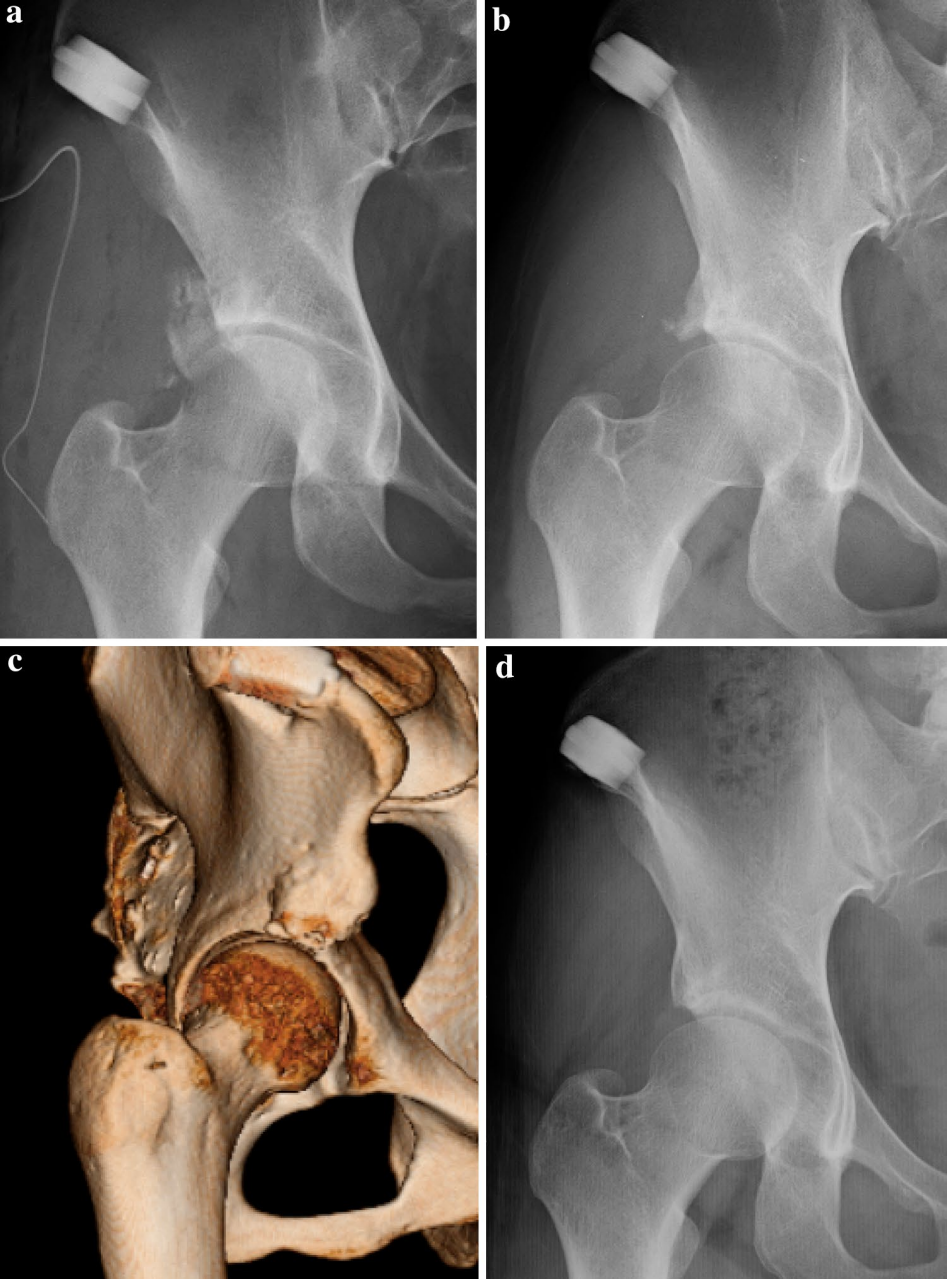
**a** Pelvic AP radiograph showing improved coverage of the acetabulum with a shelf graft just after surgery. LCE angle was 41°. **b** Pelvic AP radiograph showing proper resorption and remodeling of the shelf graft at 1 year after surgery. **c** 3D image of CT showed the location of the shelf graft at 1 year after surgery. **d** At 4 years postoperative, a pelvic AP radiograph shows a LCE angle of 34°

**Fig. 5:**
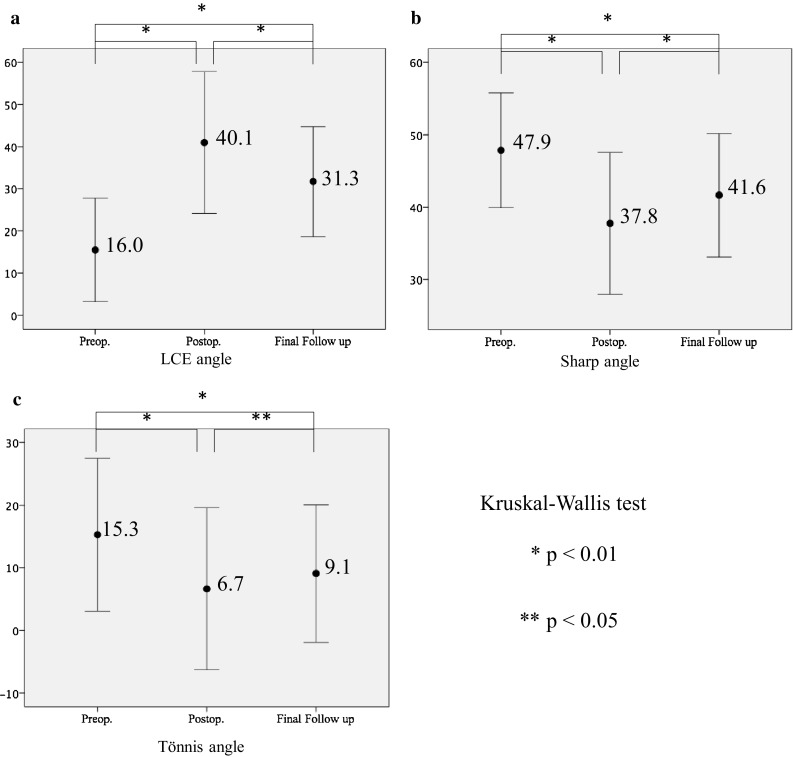
The graphic presentation of the lateral center edge (LCE) angle (**a**) the Sharp angle (**b**) and the Tönnis angle (**c**) as measured from anterior–posterior pelvis radiographs at preoperative, immediate postoperative, and at final follow-up with standard deviations (error bur). (LCE angle, preoperatively versus postoperatively versus final follow-up 16 range 7–23, versus 40.1 range 27–58, versus 30.1 range 20–41. *p* = 0.001) (Sharp angle, preoperatively versus postoperatively versus final follow-up: 47.9 range 40–58, versus 37.8 range 29–46, versus 41.6 range 28–51) (Tönnis angle, preoperatively versus postoperatively versus final follow-up: 15.3 range 1.6 ~ 34.0, versus 6.7 range − 5.0 to 21.0, versus 9.1 range − 1.0 to 19.0)

The radiographic changes including the mean LCE angle, Sharp angle and Tönnis angle are detailed in Figs. 4 and 5. The mean LCE angle significantly increased postoperatively from preoperative assessment, but partially decreased at final follow-up. The mean Sharp angle significantly decreased postoperatively from preoperative assessment (*p* = 0.001), but partially increased at final follow-up (*p* = 0.001). The mean Tönnis angle significantly decreased postoperatively from preoperative assessment (*p* = 0.001), but slightly increased at final follow-up (*p* = 0.016) (Figs. 4, 5).

### Patient-Reported Outcome (PRO) Scores

**Fig. 6:**
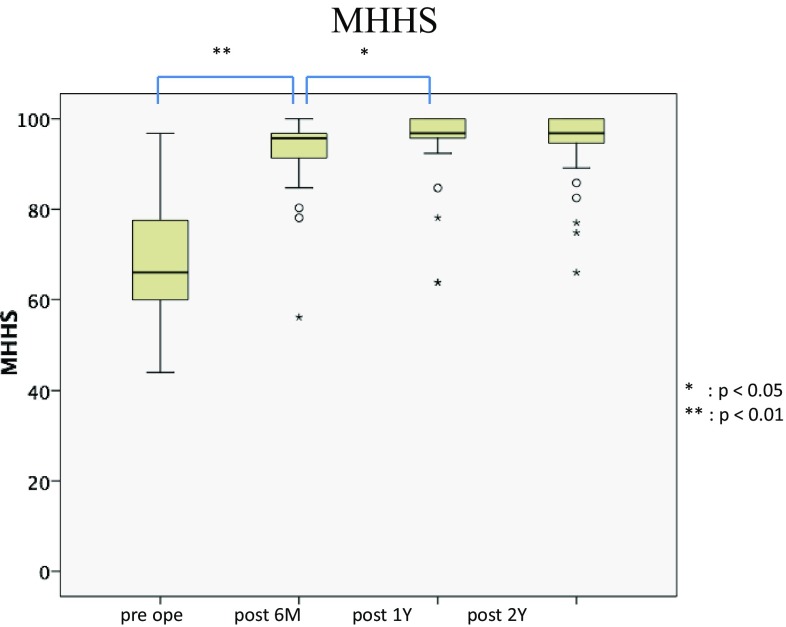
The mean MHHS significantly improved from 68.4 ± 14.3 (range 44.0–96.8) preoperatively to 92.5 ± 8.7 (range 56.1–100, *p* = 0.001) at 6 months, 94.0 ± 9.1 (range 63.8–100, *p* = 0.001) at 12 months and 94.5 ± 8.5 (range 66–100, *p* = 0.001) at final follow-up. Wilcoxon signed-rank test. *p < 0.01 **p < 0.05

**Fig. 7:**
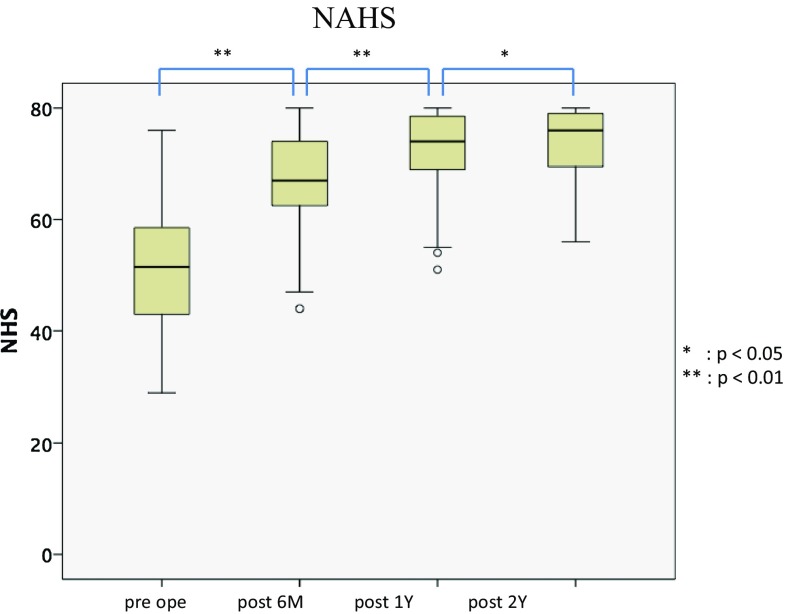
The mean NAHS also significantly improved from 51.3 ± 11.9 (range 29–76) preoperatively to 66.9 ± 9.5 (range 44–80, *p* = 0.001) at 6 months, 71.9 ± 8.1 (range 51–80, *p* = 0.001) at 12 months and 73.0 ± 7.4 (range 56–80, *p* = 0.001) at final follow-up. Wilcoxon signed-rank test. *p < 0.01 **p < 0.05

**Fig. 8:**
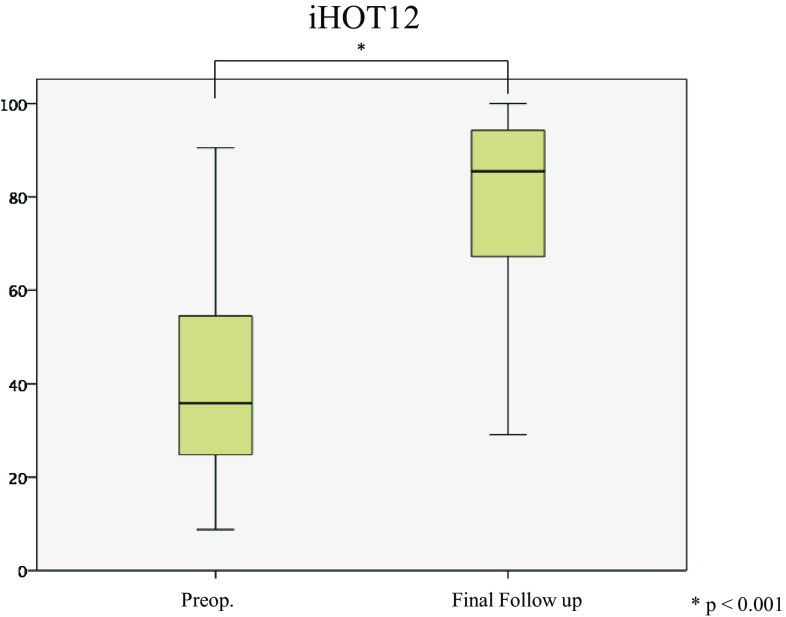
The mean iHOT-12 also significantly improved from 39.9 ± 22.3 (range 8.8–90.5) preoperatively to 78.6 ± 21.6 (range 29.1–100) at final follow-up (Fig. 7)

The mean MHHS significantly improved at 6 months and from 1 to 2 years after surgery (Fig. 6). Similarly, the mean NAHS significantly improved at 6 months, 1 year and 2 years after surgery (Fig. 7). The mean iHOT12 also significantly improved from preoperatively to at final follow-up (*p* < 0.001) (Fig. 8).

Two patients required subsequent surgery in the form of one revision arthroscopy (arthroscopic lysis of adhesions) and one conversion to total hip arthroplasty.

### Inter- and intra-observer reliability

Inter- and intra-observer reliability analyses of the radiographic measurements were assessed. The inter-observer/intra-observer ICCs of the CE angle was 0.738/0.967. The inter-observer/ intra-observer ICCs of Tönnis angle, Sharp angle, and FNS angle were 0.695/0.857, 0.847/0.42 and 0.774/0.932, respectively. The inter-observer/intra-observer reliability of the alpha angle, VCA angle and postope CE angle were 0.738 / 0.506, 0.546/0.966 and 0.556/0.909, respectively. Finally, measurements by a single observer (H.U) were utilized for further analysis.

### Return to play sports-related activity

**Fig. 9:**
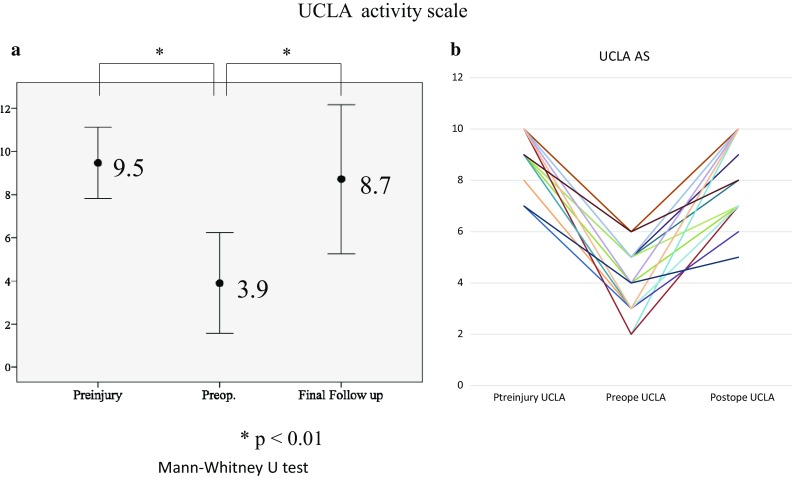
The mean University of California, Los Angeles (UCLA-AS) score (**a**) The mean UCLA-AS score significantly decreased from 9.5 (range 7–19) preinjury to 3.9 (range 2–6) preoperatively and improved to 8.7 at the final follow-up (*p* = 0.001) and UCLA-AS activity scale in each patient (**b**)

Patients reported favorable outcomes with the return to sports. 29 of 32 patients were able to return to sports-related activity. Three patients were unable to return to sports activity altogether. Of the latter, one patient experienced progressive knee osteoarthritis and one had multidirectional shoulder instability. One patient opted out of participation in sports by choice and reported no discomfort. The mean period from surgery to return to practice was 9 ± 3.5 months (range 5–18). The mean UCLA-AS score significantly decreased from preinjury to preoperatively and improved from 3.9 preoperatively to 8.7 at the final follow-up (*p* = 0.001) (Fig. 9a). The UCLA-AS score in 11 of 32 active patients decreased from preinjury to the final follow-up. The UCLA-AS score in 19 of 32 at final follow-up improved to the preinjury level. Two of 32 patients reported UCLA-AS scores above preinjury level (Fig. 9b).

### Postoperative complication

A transient lateral femoral cutaneous nerve (LFCN) neuropraxia occurred in two patients. A fracture of the shelf graft was observed in a 15-year-old Judo player. He returned to Judo at 3 months after surgery without physician’s permission. He did not require shelf graft refixation because the fracture site was at the midportion of shelf graft with residual coverage.

## Discussion

The most important findings of present study are good clinical outcomes and high rate of return to play and level of athletic function after endscopic shelf acetabuloplasty in active patients.

Rotational acetabular osteotomy (RAO) is an option for treating patients with DDH. Several studies demonstrate long-term clinical outcomes following RAO with 77–80% of those patients defined as not progressing to osteoarthritis stage III (Classification of osteoarthrosis of the hip joint adovocated by the Japanese Orthopaedic Association) [18, 23]. While the literature exists for outcomes following RAO in the general population, there is a paucity of studies specified to return to sports activity after RAO in athletes.

Periacetabular osteotomy (PAO) is another popular procedure for treating patients with DDH in the absence of osteoarthritis. There are currently two studies demonstrating clinical outcomes and sports activity following PAO. Van Bergayk et al. reported 19 of 21 patients were able to participate in sports following PAO. Recently, Ettinger et al. in other series of 77 patients undergoing PAO have shown the mean UCLA-AS score significantly improved from 4.8 preoperatively to 7.7. However, they did not mention the prevalence of return to play. At final follow-up, the UCLA-AS score in our study decreased from preinjury level in 11 active patients (Fig. 9a), returned to preinjury level in 19, and increased beyond preinjury level in 2. Despite partial resorption of the shelf bone graft observed in this study, the LCE angle representative of lateral coverage remained significantly increased at minimum 2 years of follow-up. Shelf resorption has been known to occur on occasion and has been attributed to high bone graft placement lacking sufficient desirable pressure against the underlying femoral head and interposed capsule [28]. Evidence of graft resorption, even severe, may not necessarily dictate clinical failure. This finding was reported in a long-term study of open shelf acetabuloplasty and was attributed to the residual improvement of static lateral coverage [28]. It may be prudent to use an adequately long bone graft to enable sufficient anterolateral coverage (perhaps to a LCE angle near 30°–40° on intra-operative fluoroscopic examination) in anticipation of some partial resorption.

Open shelf acetabuloplasty, without intra-articular surgery, has also been performed for the treatment of DDH. Some studies revealed that labral tearing could adversely influence the clinical outcome of shelf acetabuloplasty [1, 10]. Unlike PAO and RAO which can reposition hyaline cartilage into weight-bearing locations via acetabular reorientation, shelf acetabuloplasty relies upon capsular metaplasia into fibrocartilage. Furthermore, the shelf procedure, using a relatively uniplanar bone graft, provides more focal lateral than anterior coverage. Nevertheless, other studies demonstrated that shelf acetabuloplasty without any intra-articular procedures can provide satisfactory long-term results [12, 21]. Endoscopic shelf acetabuloplasty is minimally invasive with promising short-term outcomes in a general population with DDH; it is now routinely used for mild or moderate DDH and for cases of failed isolated hip arthroscopy in borderline DDH in our clinical practice. The findings of this study suggest that endoscopic shelf acetabuloplasty may be beneficial for treating active patients with hip dysplasia. However, endoscopic shelf acetabuloplasty is technically demanding and may best be performed by surgeons experienced in hip arthroscopy, somewhat limiting its utility.

Some minor complications such as lateral femoral cutaneous nerve neuropraxia and fracture of shelf graft were found in this study. This procedure required to extend MAP distally to insert shelf graft. In these two cases with lateral femoral cutaneous nerve palsy, a Tinel-like sign was produced upon tapping the MAP incision. To avoid LFCN palsy, we prefer the modified MAP which is slightly lateral to the classic MAP. Fracture of shelf graft could occur both intraoperatively and postoperatively. Patient compliance with postoperative rehabilitation including avoidance of premature return to sport may lessen this complication, as well as adhesive capsulitis.

This study has several limitations. This was a retrospective short-term case series without a control group. Isolated hip arthroscopic labral repair might be considered as a control group, but recent studies suggest that moderate dysplasia patients cannot benefit from isolated hip arthroscopic procedures without a PAO or shelf procedure, preventing clinical equipoise. A mid- to long-term prospective randomized study is desirable to collect more information regarding shelf graft remodeling and durability of outcomes. VCA angle was measured, however, given the variance of pelvic positioning on false profile views, and it is difficult to measure the VCA angle precisely. Some studies have shown that the VCA angle is not reliable to determine accurate anterior coverage of the acetabulum. Another limitation is the lack of a strict definition of an athlete, one of our study inclusion criteria. We did not limit the study to stratify patients into professional and/or elite athlete groupings but chose to investigate all patients with self-reported competitive athletic endeavors of various aforementioned types. Although the UCLA-AS was captured as a measure of sports-related activity, a limitation is the absence of data on frequency and duration of resumed athletic endeavor.

The clinical significance of this study is evidence-based support for the addition of a minimally invasive option to open acetabular reorientation osteotomy for not only sedentary but athletic patients with symptomatic mild to moderate dysplasia. In practice, dysplasia with LCEA between approximately 15°–20°, perhaps too severe for predictably good outcomes from hip arthroscopy alone, may still be candidates for endoscopic shelf acetabuloplasty adding bony support along with the benefits of concurrent hip arthroscopy to treat coexistent intra-capsular pathology.

## Conclusion

Endoscopic shelf acetabuloplasty provides promising clinical outcomes and return to sports-related activity for active patients with DDH.
